# SP6616 as a new Kv2.1 channel inhibitor efficiently promotes *β*-cell survival involving both PKC/Erk1/2 and CaM/PI3K/Akt signaling pathways

**DOI:** 10.1038/cddis.2016.119

**Published:** 2016-05-05

**Authors:** T T Zhou, L L Quan, L P Chen, T Du, K X Sun, J C Zhang, L Yu, Y Li, P Wan, L L Chen, B H Jiang, L H Hu, J Chen, X Shen

**Affiliations:** 1 CAS Key Laboratory of Receptor Research, 3th Department of Pharmacology, Shanghai Institute of Materia Medica, Chinese Academy of Sciences, Shanghai, China; 2 University of Chinese Academy of Sciences, Beijing, China; 3 College of Life and Environmental Sciences, Shanghai Normal University, Shanghai, China

## Abstract

Kv2.1 as a voltage-gated potassium (Kv) channel subunit has a pivotal role in the regulation of glucose-stimulated insulin secretion (GSIS) and pancreatic *β*-cell apoptosis, and is believed to be a promising target for anti-diabetic drug discovery, although the mechanism underlying the Kv2.1-mediated *β*-cell apoptosis is obscure. Here, the small molecular compound, ethyl 5-(3-ethoxy-4-methoxyphenyl)-2-(4-hydroxy-3-methoxybenzylidene)-7-methyl-3-oxo-2,3-dihydro-5H-[1,3]thiazolo[3,2–a]pyrimidine-6-carboxylate (SP6616) was discovered to be a new Kv2.1 inhibitor. It was effective in both promoting GSIS and protecting *β* cells from apoptosis. Evaluation of SP6616 on either high-fat diet combined with streptozocin-induced type 2 diabetic mice or *db/db* mice further verified its efficacy in the amelioration of *β*-cell dysfunction and glucose homeostasis. SP6616 treatment efficiently increased serum insulin level, restored *β*-cell mass, decreased fasting blood glucose and glycated hemoglobin levels, and improved oral glucose tolerance. Mechanism study indicated that the promotion of SP6616 on *β*-cell survival was tightly linked to its regulation against both protein kinases C (PKC)/extracellular-regulated protein kinases 1/2 (Erk1/2) and calmodulin(CaM)/phosphatidylinositol 3-kinase(PI3K)/serine/threonine-specific protein kinase (Akt) signaling pathways. To our knowledge, this may be the first report on the underlying pathway responsible for the Kv2.1-mediated *β*-cell protection. In addition, our study has also highlighted the potential of SP6616 in the treatment of type 2 diabetes.

Type 2 diabetes mellitus (T2DM) is a chronic, complex and multifactorial metabolic disorder mainly characterized by hyperglycemia with insulin resistance and deficiency. T2DM has become a serious global health problem bringing heavy burdens to societies.^1^ Currently, a series of anti-T2DM drugs are being clinically used, but their existing side effects are still triggering the urgent need for novel agents in the treatment of this disease.^1^

Recently, accumulating evidence has revealed that pancreatic *β*-cell dysfunctions including glucose-stimulated insulin secretion (GSIS) defect and *β*-cell mass loss are major determinants for the progression from prediabetes with normoglycemia to diabetes with hyperglycemia, and the result that insulin resistance in prediabetes needs compensatory insulin hypersecretion likely leads to a progressive decline in islet *β*-cell function.^2^ Therefore, an ideal strategy for T2DM treatment is to improve pancreatic *β*-cell function.^1, 2^

Numerous electrical signaling systems including K^+^, Na^+^, Ca^2+^ and Cl^−^ fluxes across *β*-cell membranes have been determined to participate in the function and/or survival of pancreatic *β* cells.^3^ There are three major potassium fluxes in *β* cells, including K^+^ effluxes regulated by voltage-gated K^+^ (Kvs) or ATP-sensitive K^+^ (K_ATP_) channel and calcium-activated potassium channel (KCa^2+^).^3^ Kv2.1 as a voltage-gated potassium (Kv) family member accounts for the majority of Kv currents in both rodent and human and negatively regulates GSIS.^4^ In *β* cells, the ATP derived from glucose metabolism efficiently depolarizes *β* cells leading to the opening of voltage-gated ion channels.^3^ Activated K^+^ currents produce the repolarization of *β*-cell action potential resulting in the shutdown of voltage-dependent Ca^2+^ channels (VDCCs), abolishment of VDCC-mediated Ca^2+^ influx and blockage of insulin secretion.^3, 5^

Reports have demonstrated that Kv2.1 signaling regulation is involved in the apoptosis processes of neuron and *β* cells.^6, 7^ For example, Kv2.1 overexpression activates the mitochondrial or ER stress-induced apoptosis^6^ and elevates the sensitivity of cells to apoptotic factors,^7^ whereas transient expression of Kv2.1 function-deficient mutant avoids neuronal apoptosis.^7^ Therefore, Kv2.1 channel is crucial to insulin secretion and/or *β*-cell apoptosis, and Kv2.1 inhibitors function potently in the promotion of insulin secretion and/or *β*-cell protection,^8, 9, 10^ although the mechanisms underlying the regulation of *β*-cell protection still remain unclear.

Here, the small molecule, ethyl 5-(3-ethoxy-4-methoxyphenyl)-2-(4-hydroxy-3-methoxybenzylidene)-7-methyl-3-oxo-2,3-dihydro-5H-[1,3]thiazolo[3,2–a]pyrimidine-6-carboxylate (SP6616, Figure 1a) was found to be a new Kv2.1 inhibitor. It was capable of both promoting GSIS and protecting *β* cells from apoptosis. Protein kinases C (PKC)/extracellular-regulated protein kinases 1/2 (Erk1/2) and calmodulin (CaM)/phosphatidylinositol 3-kinase (PI3K)/serine/threonine-specific protein kinase (Akt) pathways were first determined to be implicated in the Kv2.1-mediated *β*-cell protection. Moreover, assays on T2DM model mice high-fat diet (HFD)/streptozocin (STZ) and *db/db* demonstrated that SP6616 efficiently ameliorated *β*-cell dysfunction and improved glucose homeostasis, further highlighting the potential of SP6616 in the treatment of T2DM.

## Results

### SP6616 is a Kv2.1 inhibitor

#### SP6616 inhibited membrane potential in CHO-Kv2.1 cells

**Figure 1:**
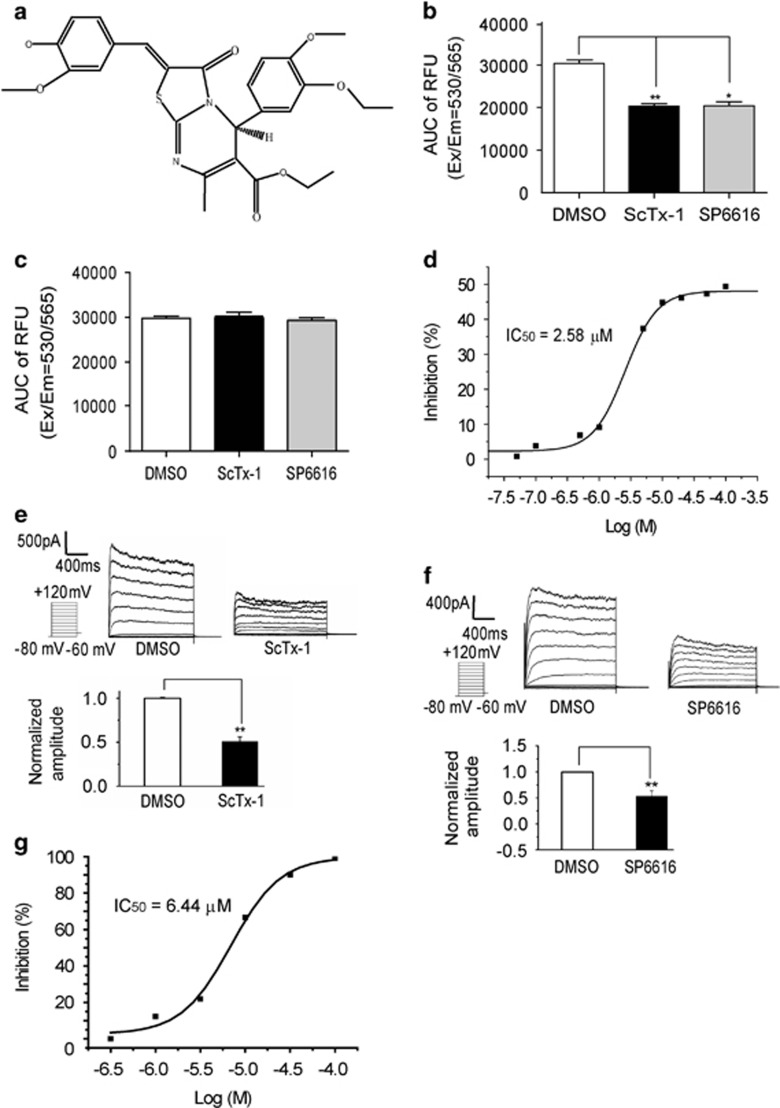
SP6616 is an inhibitor of Kv2.1 channel. (**a**) Chemical structure of SP6616. (**b**) CHO-Kv2.1 cells were incubated with 20 *μ*M SP6616 or 100 nM ScTx-1 and membrane potential dye for 30 min, and then signal was recorded. These data were analyzed and shown as AUC. (**c**) Membrane potential assay in CHO cells was conducted as described in **b**. (**d**) Fifty percent inhibitive concentration (IC_50_, 2.58 *μ*M) of SP6616 evaluated on the membrane potential assay. (**e**) Voltage-dependent outward K^+^ currents were recorded in CHO-Kv2.1 cells by whole-cell patch clamp from −80 mV to +120 mV. ScTx-1 (100 nM) effectively reduced K^+^ current amplitude. (**f**) SP6616 (10 *μ*M) inhibited K^+^ current amplitude in the whole-cell patch clamp assay. (**g**) IC_50_ (6.44 *μ*M) of SP6616 was evaluated on whole-cell patch clamp technique. All data were obtained from three independent experiments and shown as means±S.E.M. (*P<0.05, **P<0.01)

Given that the membrane potential-sensitive fluorescent dye is powerful for screening regulators of ion channels,^11^ the membrane potential (FLIPR membrane potential assay kit) based platform by FlexStationII384 was at first applied to screen Kv2.1 inhibitor candidates against the lab compound library. As shown in Figure 1b, Kv2.1 inhibitor ScTx-1 (stromatoxin-1,100 nM)^12^ obviously inhibited the membrane potential in CHO-Kv2.1 cells, indicating the efficacy of this platform in screening Kv2.1 inhibitor candidates.

Accordingly, SP6616 was discovered to be active in inhibiting membrane potential in CHO-Kv2.1 cells (Figure 1b) by IC_50_ at 2.58 *μ*M (Figure 1d). Moreover, the result that neither SP6616 (20 *μ*M) nor ScTx-1 (100 nM) inhibited membrane potential in normal CHO cells further confirmed the inhibition of SP6616 against Kv2.1 channel (Figure 1c). In addition, SP6616 was also found to inhibit Kv2.2 channel by IC_50_ at 13.48 *μ*M (Supplementary Figure 1) in CHO cells transfected with pcDNA3.1a-Kv2.2, this result thus indicated the slightly preferred selectivity of SP6616 against Kv2.1 over Kv2.2.

#### Patch clamp assay confirmed SP6616 inhibition against Kv2.1 channel

To verify SP6616 inhibition against Kv2.1 channel, the classical whole-cell patch clamp assay was performed in CHO-Kv2.1 cells. The results indicated that SP6616 inhibited Kv2.1 channel by IC_50_ at 6.44 *μ*M (Figures 1f and g), in which ScTx-1 (100 nM) was used as a positive control (Figure 1e).

Therefore, all results have determined that SP6616 was a Kv2 inhibitor with slight selectivity against Kv2.1 over Kv2.2.

### SP6616 improves *β*-cell dysfunction in a Kv2.1-dependent manner

Given that Kv2.1 inhibition mediates potently in ameliorating pancreatic *β*-cell dysfunction,^6, 9^ the effects of SP6616 on GSIS and *β*-cell survival were investigated in INS-832/13 cells.

#### SP6616 promoted GSIS

**Figure 2:**
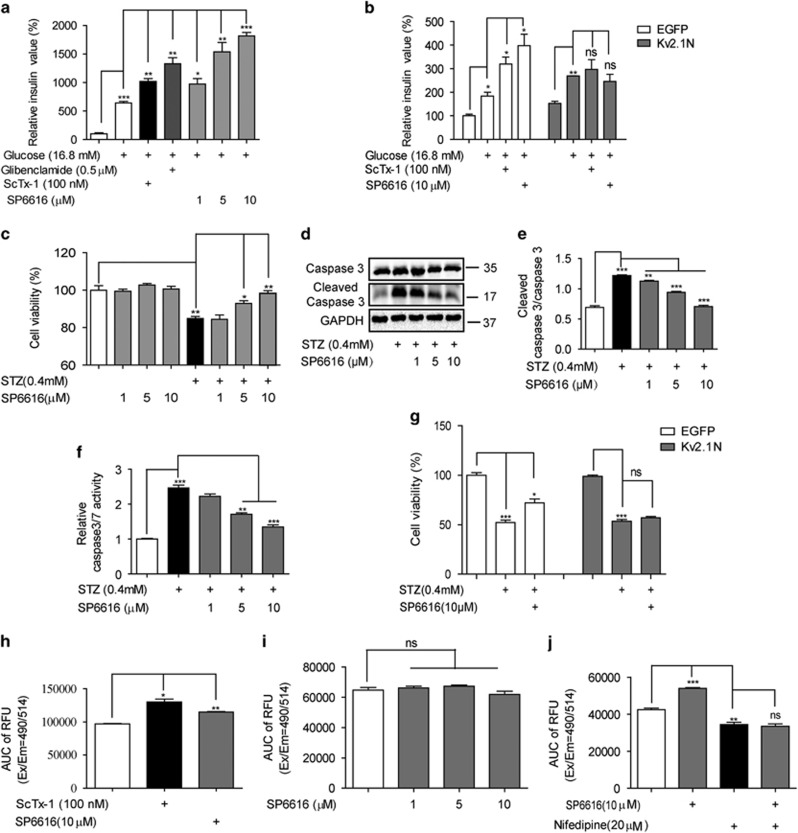
SP6616 improves pancreatic *β*-cell dysfunction by inhibiting Kv2.1 channel. (**a**) After 2-h incubation with glucose-free KRB buffer, INS-832/13 cells were incubated with SP6616 (1, 5, 10 *μ*M), ScTx-1 (100 nM) or glibenclamide (0.5 *μ*M) in the presence of 16.8 mM glucose in KRB buffer, and insulin secretion was then detected by AlphaLISA insulin kit. (**b**) INS-832/13 cells were transfected with Kv2.1N or EGFP (control), and incubated with glucose-free KRB buffer for 2 h. The cells were stimulated with SP6616 (10 *μ*M) or ScTx-1 (100 nM) in KRB buffer with 16.8 mM glucose, and insulin secretion was detected. (**c**) INS-832/13 cells were incubated with different concentrations of SP6616 (1, 5, 10 *μ*M) in the absence or presence of STZ (0.4 mM) for 24 h, and then MTT assay was conducted. (**d**) INS-832/13 cells were treated with SP6616 (1, 5, 10 *μ*M) and STZ (0.4 mM) for 8 h, and the cell lysate was then analyzed by western blot assay using caspase 3 antibody. (**e**) Relative protein levels of cleaved caspase 3/caspase 3 in **d**. (**f**) INS-832/13 cells were treated with SP6616 (1, 5, 10 *μ*M) and STZ (0.4 mM) for 8 h, and then caspase 3/7 activity was detected. (**g**) INS-832/13 cells were transfected with Kv2.1N or EGFP, and incubated with SP6616 (10 *μ*M) and STZ (0.4 mM) for 24 h, followed by MTT assay. (**h**) Intracellular Ca^2+^ level in INS-832/13 cells was monitored by Fluo-8 AM fluorescence dye. The cells were pre-incubated in KRB buffer for 2 h and then the plate was loaded on FlexStationII384. The baseline fluorescence signal was measured for the first 20 s, and then stimulated with 16.8 mM glucose in the presence of SP6616 (10 *μ*M) or ScTx-1(100 nM). These data were shown as AUC of intracellular Ca^2+^ change. (**i**) The intracellular Ca^2+^ assay was conducted as (**h**) in calcium-free HBSS buffer. (**j**) The intracellular Ca^2+^ assay involving nifedipine was conducted as **h**. All data were obtained from three independent experiments and presented as means±S.E.M. (*P<0.05, **P<0.01, ***P<0.001; ns, no significance)

GSIS assay was conducted relating to the effect of SP6616 on insulin secretion. As shown in Figure 2a (ScTx-1 and glibenclamide as positive controls), SP6616 dose-dependently activated insulin secretion in response to high concentration of glucose (16.8 mM) stimulation.

To verify the dependency of Kv2.1 inhibition for SP6616-potentiated GSIS, the dominant-negative mutant of Kv2.1 (Kv2.1N)^9, 10^ involved assay was performed. As shown in Figure 2b, transfection of Kv2.1N caused inability of SP6616 or ScTx-1 in promoting GSIS, implying that SP6616 enhanced GSIS in a Kv2.1-dependent manner.

#### SP6616 protected *β* cells from STZ-induced apoptosis

Next, we investigated the potential protection of SP6616 against *β*-cell apoptosis by 3-(4,5-dimethylthiazol-2-yl)-2,5-diphenyltetrazolium bromide (MTT) assay with STZ (0.4 mM) as cell apoptosis stimulus.^13^ As shown in Figure 2c, SP6616 had no effects on *β*-cell viability but counteracted the STZ-induced cell apoptosis in INS-832/13 cells. In addition, SP6616 also exhibited activity in antagonizing the STZ-induced increases in both the protein level of cleaved caspase 3 (pro-apoptosis protein) and the activity of caspase 3/7 (Promega, Madison, WI, USA) (Figures 2d–f), further confirming that SP6616 could protect *β*-cell from apoptosis. Moreover, Kv2.1N transfection resulted in the inactivity of SP6616 in protection against STZ-induced apoptosis (Figure 2g). Thus, these results showed that SP6616 protected *β* cells from apoptosis in a Kv2.1-dependent manner.

It is noted that the published reports indicated that Kv2.1N transfection in rat islet reduced approximately 60%-outward K^+^ currents,^9, 14^ while in the current work, the effects of SP6616 were almost fully abolished in Kv2.1N-transfected cells. Such a discrepancy may be caused by the signal transduction from current blockage to insulin secretion or anti-apoptosis in cells. Similarly, such a non-linear relationship between current blockage and insulin secretion has been also reported elsewhere.^9, 10^

Taken together, SP6616 was a new Kv2.1 inhibitor with dual effects on both insulin secretion promotion and *β*-cell protection.

### Potentiation of SP6616 on GSIS links to glucose-stimulated Ca^2+^ influx

Considering that Kv channel activation can induce membrane repolarization and VDCCs closure further reducing insulin secretion and K_V_ channel inhibition heightens intracellular Ca^2+^ level and stimulates insulin secretion,^3, 5^ we next detected intracellular Ca^2+^ level mediated by SP6616 in INS-832/13 cells. As shown in Figure 2h, either ScTx-1(100 nM) or SP6616 (10 *μ*M) increased intracellular Ca^2+^ level in the presence of 16.8 mM glucose. And such an intracellular Ca^2+^ increase was blocked by depleting extracellular calcium in Hank's balanced salt solution (HBSS) buffer or by nifedipine (L-VDCC blocker)^15^ (Figures 2i and j). These results thereby revealed that SP6616-stimulated Ca^2+^ influx in response to high glucose, similar to the published K_V_ channel inhibition-mediated GSIS event.^16^

### Ca^2+^ influx/PKC/Erk1/2 and Ca^2+^ influx/CaM/PI3K/Akt pathways are responsible for SP6616-mediated *β*-cell survival

**Figure 3:**
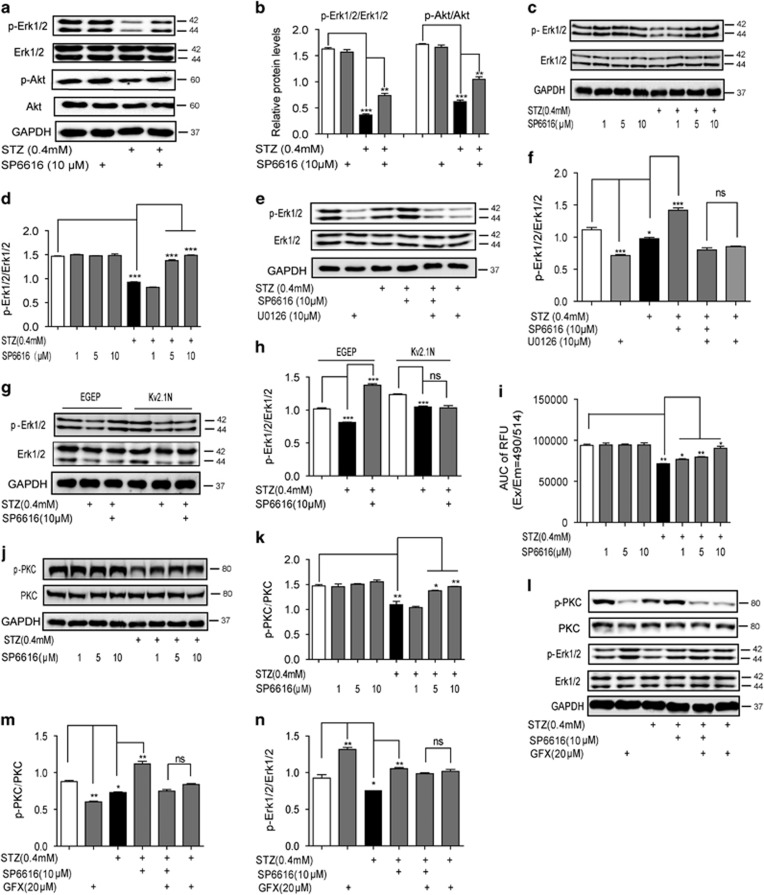
Ca^2+^ influx/PKC/Erk1/2 signaling pathway is involved in the SP6616-mediated *β*-cell protection. (**a**) INS-832/13 cells were incubated with SP6616 (10 *μ*M) in the presence or absence of STZ (0.4 mM) for 24 h, and the cell lysate was analyzed by western blot assay using the corresponding antibodies. (**b**) Relative protein levels of p-Erk1/2/Erk1/2 and p-Akt/Akt in **a**. (**c**) INS-832/13 cells were incubated with SP6616 (1, 5, 10 *μ*M) in the presence or absence of STZ (0.4 mM) for 24 h, and the cell lysate was analyzed by western blot assay using p-Erk1/2 and Erk1/2 antibodies. (**d**) Relative protein levels of p-Erk1/2/Erk1/2 in **c**. (**e**) After INS-832/13 cells were incubated with SP6616 (10 *μ*M) and STZ (0.4 mM) in the presence or absence of U0126 (10 *μ*M) for 24 h, the cell lysate was analyzed by western blot assay using p-Erk1/2 and Erk1/2 antibodies. (**f**) Relative protein levels of p-Erk1/2/Erk1/2 in **e**. (**g**) INS-832/13 cells were transfected with Kv2.1 N or EGFP, and incubated with STZ (0.4 mM) and SP6616 (10 *μ*M) or STZ alone, then the cell lysate was analyzed by western blot using p-Erk1/2 and Erk1/2 antibodies. (**h**) Relative protein levels of p-Erk1/2/Erk1/2 in **g**. (**i**) INS-832/13 cells were incubated with SP6616 (1, 5, 10 *μ*M) in the presence or absence of STZ (0.4 mM) for 24 h, then intracellular Ca^2+^ level were monitored by Fluo-8 AM fluorescence dye. Data were shown as the AUC of intracellular Ca^2+^ level. (**j**) INS-832/13 cells were incubated with SP6616 (1, 5, 10 *μ*M) in the presence or absence of STZ (0.4 mM) for 24 h, and the cell lysate was analyzed by western blot using p-PKC and PKC antibodies. (**k**) Relative protein levels of p-PKC/PKC in **j**. (**l**) INS-832/13 cells were incubated with SP6616 (10 *μ*M) and STZ (0.4 mM) in the presence or absence of GFX (20 *μ*M) for 24 h, and the cell lysate was analyzed by western blot using corresponding antibodies. (**m**) Relative protein levels of p-PKC/PKC in **l**. (**n**) Relative protein levels of p-Erk1/2/Erk1/2 in **l**. All data were obtained from three independent experiments and presented as means±S.E.M. (*P<0.05, **P<0.01, ***P<0.001; ns, no significance)

Apoptosis is the process of programmed cell death, and regulated by a variety of extrinsic factors.^17^ Although the signaling pathways in apoptosis are complicated, signaling of Erk1/2, p38, JNK, Akt or NF*κ*B is determined to be vital in apoptosis and proliferation.^17, 18^ Therefore, we examined whether SP6616-mediated *β*-cell survival was implicated in any of those five signaling pathways in INS-832/13 cells. As demonstrated in Figures 3a and b, SP6616 reversed the STZ-induced decrease of either Erk1/2 or Akt phosphorylation, but rendered no effects on p38, JNK or NF*κ*B phosphorylation (Supplementary Figure 2). Accordingly, we next investigated SP6616 protection against *β* cells by focusing on Erk1/2 and Akt signaling.

#### Erk1/2 signaling was implicated in SP6616-mediated *β*-cell protection

To investigate SP6616 regulation against Erk1/2 signaling, Erk1/2 phosphorylation (p-Erk1/2) levels under different concentrations of SP6616 were examined. As shown in Figures 3c–f, SP6616 did not affect p-Erk1/2 but reversed the STZ-induced decrease of the phosphorylation level, and such an effect was terminated by U0126 (MEK/Erk1/2 inhibitor)^13^ treatment. These results thereby showed the regulation of SP6616 against Erk1/2. Moreover, Kv2.1N transfection caused inactivity of SP6616 in antagonizing the STZ-induced decrease in p-Erk1/2 in INS-832/13 cells (Figures 3g and h), thus confirming the Kv2.1-dependent regulation of SP6616 against Erk1/2 in the cells.

### 

#### Ca^2+^ influx and PKC phosphorylation were in the upstream of Erk1/2 phosphorylation in response to SP6616 regulation

Reports demonstrated that the increase of intracellular Ca^2+^ promotes PKC phosphorylation, Ras/mitogen-activated protein kinase (MAPK) signaling cascade and Erk1/2 phosphorylation leading to the protection of cells from apoptosis.^19^ Given that SP6616-induced Ca^2+^ influx under high glucose stimulation, the regulations of SP6616 against intracellular Ca^2+^ level and PKC phosphorylation with STZ treatment were investigated. It was found that SP6616 had no effects on Ca^2+^ influx, but could reverse the STZ-induced decreases both in intracellular Ca^2+^ level (Figure 3i) and PKC phosphorylation (Figures 3j and k). These results thus indicated that SP6616 may induce Ca^2+^ influx and activate PKC to stimulate Erk1/2 phosphorylation in INS-832/13 cells. Moreover, the regulation of SP6616 against PKC/Erk1/2 pathway was further verified by the assay involving PKC pan-inhibitor GF109203X (GFX),^20^ in that GFX resisted the effects of SP6616 on Erk1/2 phosphorylation (Figures 3l–n). Interestingly, GFX itself increased p-Erk1/2 in INS-832/13 cells (Supplementary Figure 3), whereas the published results indicated that GFX exhibited no effects on p-Erk1/2 in the cells with treatment of glucose or IGF-1.^21^ We here tentatively supposed that such a discrepancy may be due to the different experimental conditions.

Taken together, Ca^2+^ influx/PKC/Erk1/2 pathway was determined to be involved in the protection of SP6616 against STZ-induced *β*-cell apoptosis.

#### SP6616 regulated Akt and its downstream effectors Forkhead box protein O1 (FoxO1), X-linked inhibitor of apoptosis protein (XIAP) and Bad

**Figure 4:**
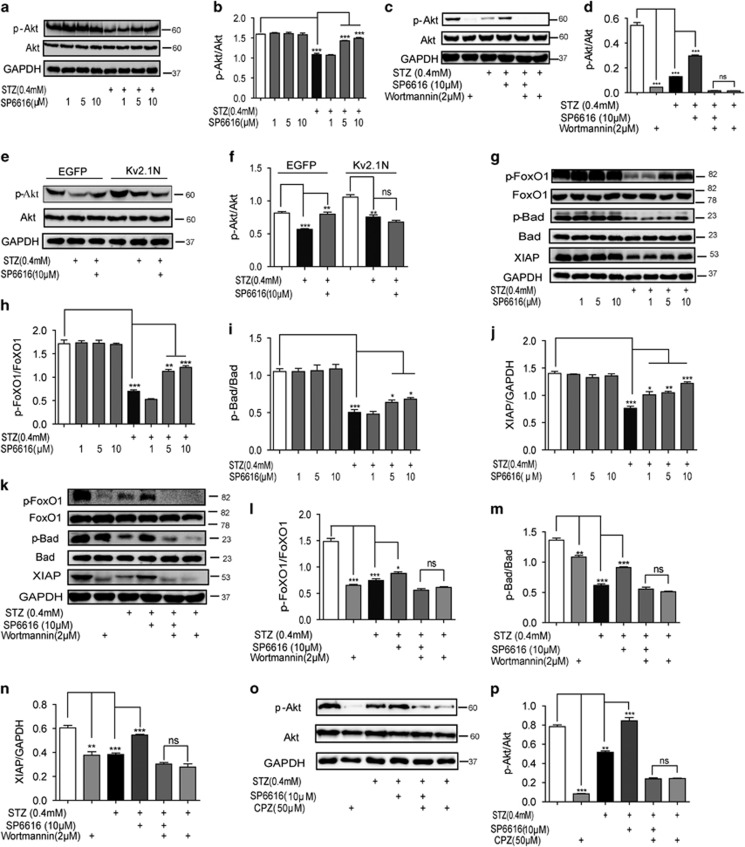
CaM/PI3K/Akt pathway is involved in the SP6616-mediated *β*-cell protection. (**a**) INS-832/13 cells were incubated with SP6616 (1, 5, 10 *μ*M) in the presence or absence of STZ (0.4 mM) for 24 h, and the cell lysate was analyzed by western blot using p-Akt and Akt antibodies. (**b**) Relative protein levels of p-Akt/Akt in **a**. (**c**) INS-832/13 cells were incubated with SP6616 (10 *μ*M) and STZ (0.4 mM) for 20 h in the presence or absence of wortmmanin (2 *μ*M) for another 4 h, and then cell lysate was analyzed by western blot using p-Akt and Akt antibodies. (**d**) Relative protein levels of p-Akt/Akt in **c**. (**e**) INS-832/13 cells were transfected with Kv2.1 N or EGFP, and incubated with STZ (0.4 mM) in the presence or absence of SP6616 (10 *μ*M), and then the cell lysate was analyzed by western blot using p-Akt and Akt antibodies. (**f**) Relative protein levels of p-Akt/Akt in **e**. (**g**) INS-832/13 cells were incubated with SP6616 (1, 5, 10 *μ*M) in the presence or absence of STZ (0.4 mM) for 24 h, and the cell lysate was analyzed by western blot using the corresponding antibodies. (**h**) Relative protein levels of p-Foxo1/Foxo1 in **g**. (**i**) Relative protein levels of p-Bad/Bad in **g**. (**j**) Relative protein levels of XIAP/GAPDH in **g**. (**k**) INS-832/13 cells were incubated with SP6616 (10 *μ*M) and STZ (0.4 mM) for 20 h in the presence or absence of wortmmanin (2 μM) for another 4 h, and then cell lysate was analyzed by western blot using the corresponding antibodies. (**l**) Relative protein levels of p-Foxo1/Foxo1 in **k**. (**m**) Relative protein levels of p-Bad/Bad in **k**. (**n**) Relative protein levels of XIAP/GAPDH in **k**. (**o**) INS-832/13 cells were pre-incubated with SP6616 (10 *μ*M) and STZ (0.4 mM) for 23 h and then with or without stimulation of CPZ (50 *μ*M) for 1 h, finally the cell lysate was analyzed by western blot using p-Akt and Akt antibodies. (**p**) Relative protein levels of p-Akt/Akt in **o**. All data were obtained from three independent experiments and presented as means±S.E.M. (*P<0.05, **P<0.01, ***P<0.001; ns, no significance)

Next, we investigated the regulation of SP6616 against Akt signaling in INS-832/13 cells. As shown in Figures 4a and b, SP6616 had no effects on Akt phosphorylation (p-Akt) but reversed the STZ-induced decrease in p-Akt. Notably, incubation of wortmannin (PI3K inhibitor)^22^ in the cells caused the inactivity of SP6616 in recovering the STZ-reduced Akt phosphorylation (Figures 4c and d). These results thereby implied the regulation of SP6616 against Akt signaling. In addition, to determine whether the SP6616-increased p-Akt was dependent on Kv2.1 regulation, the relevant assays in Kv2.1N-transfected INS-832/13 cells were performed. As illustrated in Figures 4e and f, the ability of SP6616 in reversing the STZ-decreased p-Akt weakened, this result thereby indicated that SP6616-stimulated Akt phosphorylation in a Kv2.1-dependent manner.

Given that the effectors involved in Akt-mediated anti-apoptotic pathways mainly include FoxO1, Bad and XIAP in *β* cells,^23^ we next examined the potential regulation of SP6616 against these three downstream proteins. As shown in Figures 4g–j, SP6616 reversed the STZ-induced decreases in phosphorylated FoxO1 (p-Ser256)/Bad (p-Ser136) and protein level of XIAP. Moreover, western blot results (Figures 4k–n) showed that wortmannin treatment could block all above SP6616-induced effects, thus addressing the dependence of the regulation against Akt in the signaling.

#### Ca^2+^ influx and CaM activation were in the upstream of SP6616-stimulated Akt phosphorylation

Given that cytosolic-free calcium activates PI3K/Akt pathway through regulation of CaM^24, 25^ and SP6616-induced Ca^2+^ influx, we next investigated whether CaM stimulation linked Kv2.1 inhibition to PI3K/Akt pathway activation in INS-832/13 cells. As indicated in Figures 4o and p, incubation of CaM antagonist chlorpromazine (CPZ)^26^ caused almost the inactivity of SP6616 in reversing the STZ-reduced Akt phosphorylation.

Therefore, all results showed that both Ca^2+^ influx/PKC/Erk1/2 and Ca^2+^ influx/CaM/PI3K/Akt signaling pathways were involved in SP6616-mediated *β*-cell protection.

#### PKC/Erk1/2 and CaM/PI3K/Akt pathways were required in parallel for SP6616 protection against *β* cells

**Figure 5:**
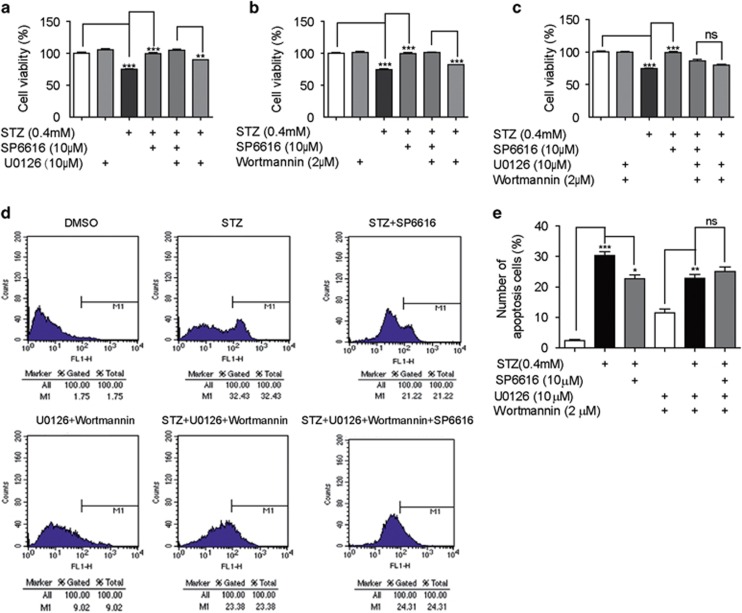
PKC/Erk1/2 and CaM/PI3K/Akt pathways are required in parallel for the protection of SP6616 against *β* cells. (**a**) INS-832/13 cells were incubated with SP6616 (10 *μ*M) and STZ (0.4 mM) in the presence or absence of U0126 (10 *μ*M) for 24 h, then MTT assay was conducted. (**b**) INS-832/13 cells were incubated with SP6616 (10 *μ*M) and STZ (0.4 mM) for 20 h in the presence or absence of wortmmanin (2 *μ*M) for another 4 h, and then MTT assay was conducted. (**c**) INS-832/13 cells were incubated with the corresponding compounds (the same concentrations and incubation time as **a** and **b**), and MTT assay was conducted. (**d**) INS-832/13 cells treated as **c** were stained with Annexin V-FITC, and then Annexin V-FITC positive INS-832/13 cells were determined by flow cytometry. (**e**) The percentage of cell apoptosis was determined by flow cytometry from three independent experiments. All data were obtained from three independent experiments and presented as means±S.E.M. (*P<0.05, **P<0.01, ***P<0.001; ns, no significance)

As either PKC/Erk1/2 or CaM/PI3K/Akt pathway has been determined to be involved in the protection of SP6616 against *β*-cell apoptosis, we next examined whether these two signaling pathways were required for the SP6616-induced protection against the cells. MTT assay was at first carried out. As indicated in Figures 5a–c, treatment with either U0126 (Figure 5a) or wortmannin (Figure 5b) in the cells failed to deprive SP6616 of its capability in protecting cell viability against the STZ-induced apoptosis. However, co-incubation of both U0126 and wortmannin (Figure 5c) in the cells almost blocked such SP6616-induced protection. Moreover, the results in quantitative evaluation of apoptosis by Annexin V-FITC staining further confirmed that SP6616 attenuated STZ-induced apoptosis and co-incubation of both U0126 and wortmannin in the cells could block this attenuation (Figures 5d and e).

Therefore, all results implied that PKC/Erk1/2 and CaM/PI3K/Akt pathways were required in parallel for the SP6616-induced *β*-cell survival promotion as summarized in Figure 8e.

### SP6616 ameliorates hyperglycemia in type 2 diabetic model mice

**Figure 6:**
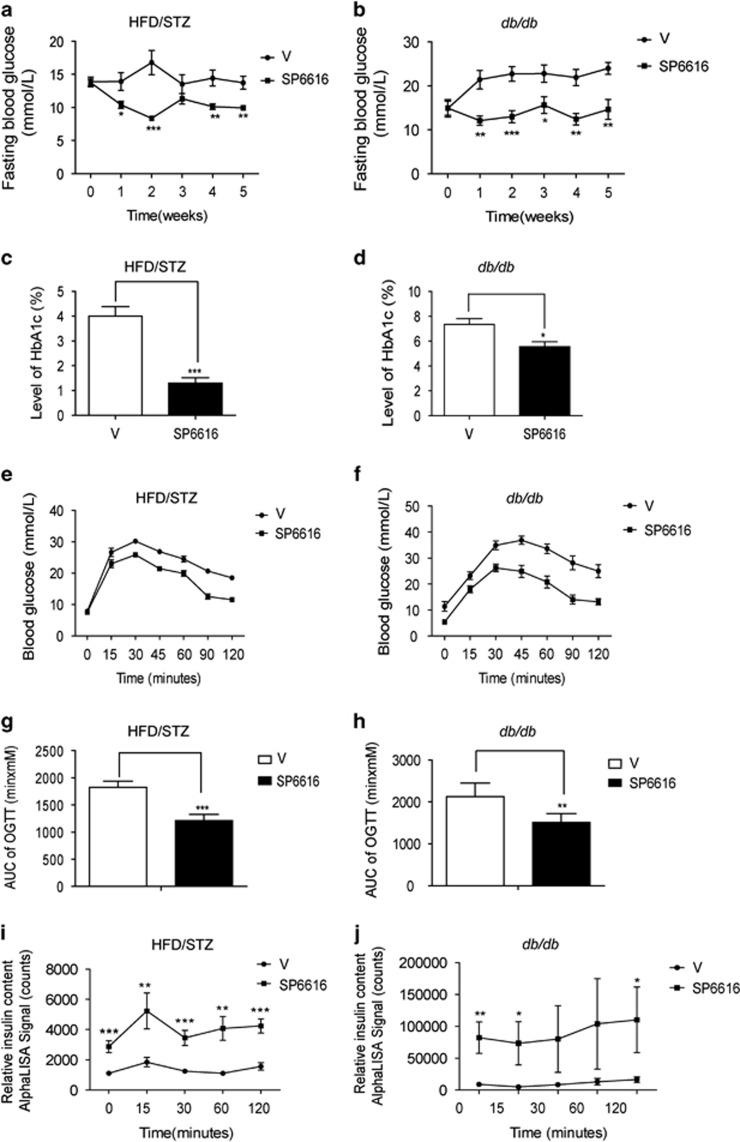
SP6616 effectively ameliorates hyperglycemia in type 2 diabetic model mice. Fasting serum glucose level was detected weekly in (**a**) HFD/STZ and (**b**) *db/db* mice with treatment of SP6616 (50 mg/kg/day) (*n*=8) (black circles, Vehicle group (V); black squares, SP6616 group (SP6616)). Plasma HbA1c level in (**c**) HFD/STZ and (**d**) *db/db* mice after treatment with SP6616 for 5 weeks was determined. OGTT was performed in (**e**) HFD/STZ and (**f**) *db/db* mice with SP6616 treatment (*n*=8). (**g**) AUC result of OGTT in **e**. (**h**) AUC result of OGTT in **f**. (**i**) Serum insulin concentration was determined during OGTT of (**e**) by AlphaLISA insulin kit. (**j**) Serum insulin concentration was determined during OGTT of **f**. All data were presented as means±S.E.M. (*P<0.05, **P<0.01, ***P<0.01)

As SP6616 has been determined to promote GSIS and *β*-cell survival, we next examined its activity in amelioration of hyperglycemia on type 2 diabetic model mice. In the assay, the model mice HFD/STZ and *db/db* were applied, and the male mice were administered with SP6616 (50 mg/kg/day) or vehicle by i.p. injection for 5 weeks. The results showed that SP6616 administration lowered the fasting blood glucose and glycated hemoglobin (HbA1c) levels (Figures 6a–d), and improved the glucose tolerance (Figures 6e–h) and insulin secretion during oral glucose tolerance test (OGTT; Figures 6i and j) in both models. Therefore, all results suggested that SP6616 effectively ameliorated hyperglycemia in type 2 diabetic mice.

### SP6616 promotes insulin secretion and *β*-cell mass in type 2 diabetic model mice

**Figure 7:**
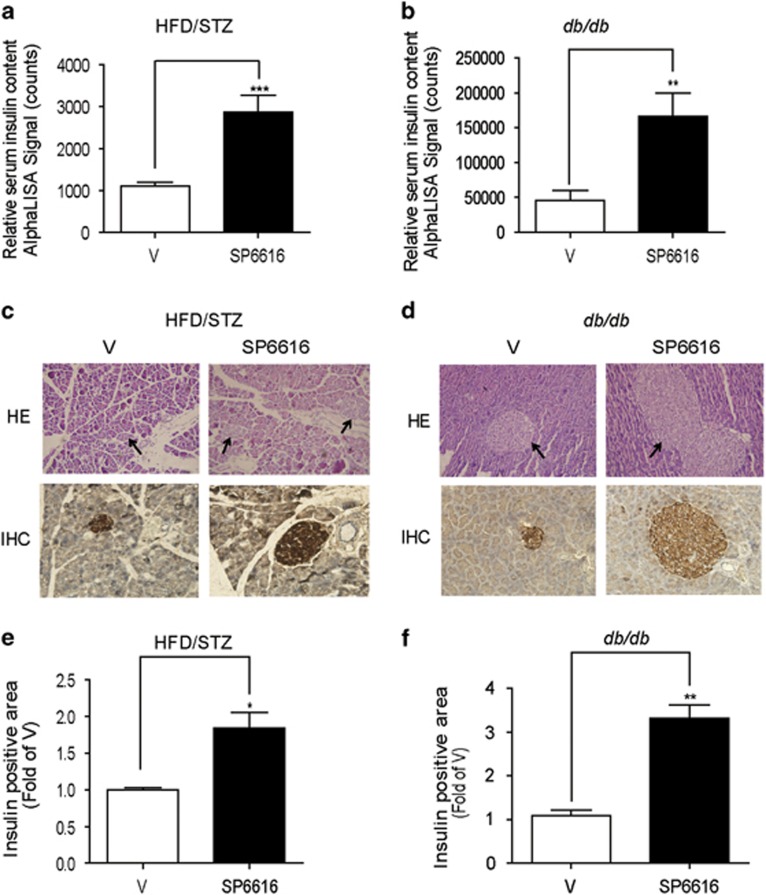
SP6616 promotes insulin secretion and *β*-cell mass in type 2 diabetic model mice. Fasting serum insulin level was detected by AlphaLISA insulin kit in (**a**) HFD/STZ (**b**) *db/db* mice after the animals were killed (*n*=8). Morphology (HE staining) and insulin immunohistochemistry (IHC) of pancreatic *β* cells in (**c**) HFD/STZ and (**d**) *db/db* mice were examined after SP6616 (50 mg/kg/day) treatment for 5 weeks. Arrows pointed to islet and size bar was 100 *μ*m. (**e**) Quantification of insulin-positive islets in **c**. (**f**) Quantification of insulin-positive islets in **d**. All data were presented as means±S.E.M. (*P<0.05, **P<0.01, ***P<0.01)

Considering that SP6616 improved *β*-cell dysfunction by promoting insulin secretion and protecting *β* cell from apoptosis, we next evaluated the potential of this agent in stimulating plasma insulin content and insulin-positive islet mass in both the two diabetic mice. As expected, SP6616-treated groups possessed higher serum insulin levels (Figures 7a and b) and more insulin-positive islets compared with vehicle groups (Figures 7c–f).

### SP6616 regulates Erk1/2 and Akt signaling *in vivo*

**Figure 8:**
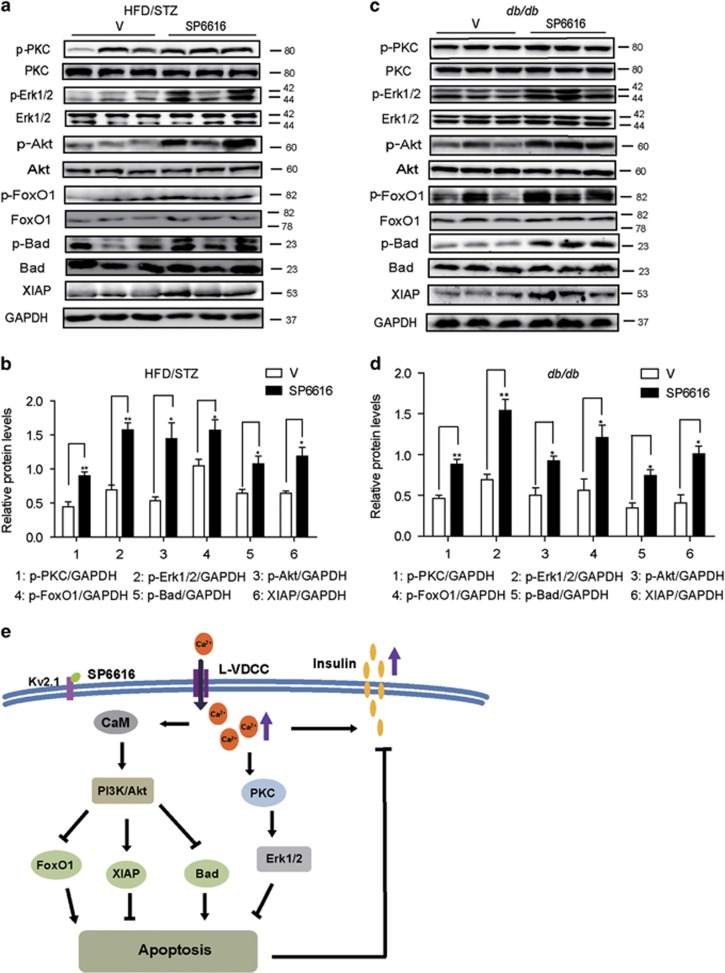
SP6616 regulates Erk1/2 and Akt signaling *in vivo*. (**a**) Pancreatic islet tissue extracts from HFD/STZ mice after SP6616 (50 mg/kg/day) treatment for 5 weeks were analyzed by western blot using the corresponding antibodies (*n*=3). (**b**) Relative protein levels in **a**. (**c**) Pancreatic islet tissue extracts from *db/db* mice after SP6616 (50 mg/kg/day) treatment were analyzed by western blot (*n*=3). (**d**) Relative protein levels in **c**. (**e**) A proposed model interpreting the involvement of PKC/Erk1/2 and CaM/PI3K/Akt signaling pathways in the SP6616-mediated *β*-cell protection. All data were presented as means±S.E.M. (*P<0.05, **P<0.01)

In view of the cell-based result that PKC/Erk1/2 and CaM/PI3K/Akt pathways were responsible for SP6616-mediated *β*-cell protection, we next evaluated SP6616 regulation against these two pathways *in vivo*. In the assay, the pancreatic tissues of both diabetic model mice were assayed by western blot against the key proteins involved in the pathways. As shown in Figures 8a–d, SP6616 administration in either model caused the increases in phosphorylated PKC, Erk1/2, Akt, FoxO1 and Bad and protein level of XIAP, totally consistent with the cell-based results, confirming the involvements of both Erk1/2 and Akt signaling in the protection of SP6616 against pancreatic *β* cells.

## Discussion

Kv2.1 channel is widely expressed in mammalian tissues including cardiomyocytes, muscles, brain and pancreatic *β* cells.^27^ As a major Kv family member, Kv2.1 channel contributes to 65–80% of the total Kv currents in human and rodent *β* cells. It has a crucial role in pancreatic *β*-cell membrane repolarization and its function.^3, 4, 9^ The fact that Kv2.1 inhibition promotes insulin secretion in response to high glucose implies the possibility in avoiding the side effect of hypoglycemia.^28, 29^ Besides, Kv2.1 also functions potently in the regulation of cell apoptosis although the underlying mechanisms have not yet been unveiled.^7^

Currently, several kinds of Kv2.1 inhibitors have been discovered. For example, peptide-type inhibitors include hanatoxin, guangxitoxin-1E, heteroscordratoxins, ScTx-1, SGTx1, syntaxin-1A, SsmTx-I and plasma gelsolin.^30, 31, 32, 33, 34, 35, 36^ Small molecular inhibitors galantamine and isoliquiritigenin block Kv2.1 currents with little data on GSIS or cell apoptosis;^37, 38^ RY796 and C-1 enhance GSIS;^8, 9^ donepezil and 48F10 abolish neuronal apoptosis.^39, 40^ Previously, we reported natural product vindoline functioned in promotion of both insulin secretion and *β*-cell protection.^10^ SP6616 is a new kind of small molecular Kv2 inhibitor with slight selectivity against Kv2.1 over Kv2.2 sharing totally different structure with the published inhibitors. Structurally, SP6616 possesses (1,3)thiazolo(3,2-a)pyrimidine scaffold whose derivatives are known to exhibit varied biological activities, including anti-viral, anti-neoplastic, anti-bacterial and anti-inflammatory.^41^ Our current work has further expanded the pharmacological applications of this kind of compound. To our knowledge, SP6616 and vindoline may be the only two small molecular Kv2.1 inhibitors able to both promote insulin secretion and survival. Moreover, SP6616 as a new Kv2.1 inhibitor effectively ameliorates *β*-cell dysfunction and improves glucose homeostasis *in vivo*. All these results have highlighted the potential of SP6616 in the treatment of type 2 diabetes.

It is accepted that activation of Kv channel can inhibit insulin secretion by inducing membrane repolarization and closure of VDCCs, and K_V_ inhibition stimulates insulin secretion.^3, 5^ Here, we found that SP6616 as a Kv2.1 inhibitor effectively stimulated GSIS by following this underlying mechanism.

Ca^2+^ is a ubiquitous cellular signaling molecule controlling a variety of cellular processes including cell survival.^42^ PKC isoform as a downstream transducer of Ca^2+^ participates in multifarious signaling pathways of biological processes including survival, proliferation, tumorigenesis and angiogenesis.^43^ Erk1/2 is an important member of the MAPK family and has a critical role in pancreatic *β* cells, particularly in the regulation of proliferation and survival.^44, 45^ An increase of intracellular Ca^2+^ can evoke PKC activation in triggering Erk1/2 stimulation.^19^ CaM, a loop-helix-loop Ca^2+^-binding protein as another downstream transducer of Ca^2+^ potently regulates multiple processes in eukaryotic cells, like proliferation and growth.^46^ Besides, increase of intracellular-free Ca^2+^ activates PI3K/Akt signaling via CaM in different cell lines.^24, 25^ PI3K/Akt pathway is known to promote survival of many cell lines, the anti-apoptotic targets of Akt signaling mainly include FoxO1, Bad and XIAP in *β* cells.^23^ FoxO1 is a transcription factor regulating cellular processes like glucose metabolism, apoptosis, cell cycle regulation and DNA damage repair.^47^ Phosphorylation of FoxO1 regulated by Akt promotes its nuclear exclusion and inhibits its pro-apoptosis function.^48^ Besides, Akt inactivates the pro-apoptotic activity of Bad by mediating the phosphorylation at Ser136.^23^ Akt has also been shown to promote cell survival by enhancing the stability of XIAP,^49^ which is one of the conserved family of IAP that suppresses apoptosis by directly binding and inhibiting caspases activity.^50^ Here, we have well determined the regulation of SP6616 against the STZ-reduced intracellular Ca^2+^ and phosphorylation levels or protein levels of the related effectors such as PKC, Erk1/2, Akt, FoxO1, Bad and XIAP both *in vitro* and *in vivo*. All results have clearly expounded the potential mechanisms underlying SP6616 protection against *β* cells. To our knowledge, PKC/Erk1/2 and CaM/PI3K/Akt may be the first reported pathways linked to the regulation of Kv2.1-mediated *β*-cell protection. Interestingly, Bcl-2 has a central role in eukaryotic cell survival by inhibiting cell death, but Bcl-2 regulation is here probably not involved in the SP6616-mediated *β*-cell protection (Supplementary Figure 4), which may be due to the insensitivity of Bcl-2 against this apoptotic event.^51^

Given that Kv2.1 channel is also highly expressed in mammalian cardiomyocytes^27^ and cardiotoxicity evaluation is vital for drug development, the potential effect of SP6616 on cardiac function in normal mice was also examined in the current work. As indicated in electrocardiography assay (Supplementary Figure 5), acute administration of SP6616 slightly prolonged QT intervals without affecting heart rates, which is consistent with the report that QT intervals are obviously prolonged without effect on heart rates in mice expressing a dominant-negative Kv2 α subunit.^52^ Our results imply that anti-diabetic drug development targeting SP6616 as a lead compound needs further investigation containing pharmacokinetics, pharmaceutics, drug toxicology and even structural modification.

In conclusion, we identified that small molecule SP6616 as a new Kv2.1 inhibitor effectively enhanced insulin secretion and protected *β* cells from apoptosis. It is determined that PKC/Erk1/2 and CaM/PI3K/Akt pathways are required in parallel for Kv2.1-mediated *β*-cell protection (Figure 8e).

## Materials and Methods

### Materials

STZ, ScTx-1, MTT, GFX were purchased from Sigma-Aldrich (St. Louis, MO, USA). U0126 and wortmannin were from Selleck Chemicals (Houston, TX, USA), CPZ from J&K Scientific (Shanghai, China) and SP6616 from commercial compound library SPECS (Zoetermeer, Netherlands). Antibodies against phospho-Akt(Ser473), Akt, phospho-Erk1/2(T202/Y204), Erk1/2, phospho-FoxO1(Ser256), FoxO1, phospho-Bad(Ser136), Bad, phospho-PKC*α*/*β*_II_ (T638/641), PKC*α*, XIAP were from Cell Signaling Technology (Danvers, MA, USA), and glyceraldehyde-3-phosphate dehydrogenase (GAPDH) from Kangcheng Biotech (Shanghai, China).

### Plasmids

pLV-EYFP-N-Kv2.1 plasmid for lentivirus-mediated stable Kv2.1 overexpression in CHO cells was constructed from pcDNA3.1a-Kv2.1 as described previously.^10^ Dominant-negative Kv2.1N subunit (Kv2.1N) plasmid was generously gifted by Professor Patrick E MacDonald (University of Toronto, Toronto, Canada).

### Cell cultures

INS-832/13 cells (kindly provided by Professor Yong Liu, Institute for Nutritional Sciences, SIBS, Chinese Academy of Sciences) were cultured in RPMI-1640 medium (Invitrogen, Grand Island, NY, USA) supplemented with 10% fetal bovine serum (FBS; Gibco, Grand Island, NY, USA), 100 U/ml penicillin and 100 mg/ml streptomycin, 10 mM HEPES, 2 mM l-glutamine, 1 mM sodium-pyruvate and 0.05 mM *β*-mercaptoethanol.

CHO-Kv2.1 cell line was established by infecting the mature lentivirus of Kv2.1 packaged in 293 T cells and cultured in Dulbecco's modified Eagle's medium (Invitrogen) with 10% FBS, 100 U/ml penicillin, 100 mg/ml streptomycin and 0.25 μg/ml puromycin. CHO cells were cultured under similar conditions except for puromycin selection.

### Membrane potential assay

Cellular membrane potential was detected by FLIPR membrane potential assay kit (Molecular Devices, Sunnyvale, CA, USA) according to the instruction manual in CHO-Kv2.1 or CHO cells. Briefly, cells were plated into 96-well microplates and incubated overnight. After treating with compounds and membrane potential dye for 30 min, the plates were loaded into FlexStationII384 (Molecular Devices), followed by injecting 20 *μ*M compounds and 100 mM KCl into the wells to generate the change of cellular membrane potential. The signals in each well were acquired for 120 s containing 20 s pre-injection basal reading at excitation wavelength of 530 nm and emission wavelength of 565 nm. These data were analyzed and shown as the area under the curve (AUC).

### Electrophysiological recording assay

The whole-cell patch clamp recordings were performed using cultured CHO-Kv2.1 cells at room temperature with Axopatch-200B amplifier (Molecular Devices) as described previously.^53^ The electrodes were pulled from borosilicate glass capillaries (1B150F-4; World Precision Instruments, Sarasota, FL, USA) by using Flaming/Brown type micropipette puller (P-97; Sutter Instrument, Novato, CA, USA). Pipettes had resistances of 3–7 MΩ when filled with a solution as following composition: 140 mM KCl, 2 mM MgCl_2_, 10 mM EGTA, 1 mM CaCl_2_, 10 mM HEPES (pH7.3). Cells were bath perfused with a solution of the following composition: 150 mM NaCl, 5 mM KCl, 0.5 mM CaCl_2_, 1.2 mM MgCl_2_, 10 mM HEPES (pH7.3). The signals were filtered at 1 kHz, digitized using a DigiData 1440 A (Molecular Devices), and analyzed with the software of pClamp 10.2 (Molecular Devices). Whole-cell currents were recorded using the protocol as follows: the holding potential was set at -80mV, and stepwise depolarized from −80 to 120 mV in 20 mV increments and then repolarized to −60 mV. Initially, we made compensation to get rid of pipette resistance during whole-cell patching, and then we made 60–80% compensation of both series resistance and capacitance of cell bodies to avoid voltage deviations (for the detailed procedures, see also Supplementary Information), especially when the current amplitude is large. For large cells, we further perform the correction offline by recording much of empty control CHO cells.^54^ Liquid junction potentials were <2 mV, which were calculated using JPCalc software.^55^

### GSIS assay

GSIS assay was carried out according to the published approach.^13^ INS-832/13 cells were plated into 24-well plates. After pre-incubated with Krebs–Ringer bicarbonate (KRB) buffer (115 mM NaCl, 5 mM KCl, 24 mM NaHCO_3_, 2.5 mM CaCl_2_, 1 mM MgCl_2_, 10 mM HEPES at pH7.2) supplemented with 0.2% bovine serum albumin for 2 h, the cells were incubated in KRB buffer containing 16.8 mM glucose and corresponding compounds for another 2 h. Supernatant of the cells was collected to detect insulin content with AlphaLISA insulin kit (PerkinElmer, Fremont, CA, USA). Insulin level was normalized with total protein content.

### MTT assay

MTT assay was performed according to previously described.^13^ INS-832/13 cells were plated into 48-well plates and incubated with different concentrations of SP6616 and STZ (0.4 mM) for 24 h (unless indication, STZ concentration was 0.4 mM and incubation time was fixed at 24 h throughout this current work).

### Flow cytometry assay

INS-832/13 cells were plated into six-well plates and incubated with corresponding compounds as indicated before collecting. Cell apoptosis was measured with Annexin V-FITC apoptosis detection kit (BD Biosciences Company, San Jose, CA, USA) following the manufacturer's protocol. The percentage of cell apoptosis was determined by flow cytometry (BD Biosciences Company).

### Intracellular Ca^2+^ assay

Intracellular Ca^2+^ measurement was performed in INS-832/13 cells incubated with calcium indicator Fluo-8 AM (2 mM) at 37 °C for 40–50 min. The relative fluorescence signals were measured for 80 s with FlexStationII384 at excitation wavelength of 490 nm and emission wavelength of 514 nm. SP6616 (1 *μ*M, 5 *μ*M, 10 *μ*M) in HBSS buffer with 16.8 mM glucose was added to the well after pre-injection basal reading. For evaluation of the SP6616-induced intracellular Ca^2+^ level change in response to STZ, different concentrations of SP6616 in HBSS buffer with 0.4 mM STZ was added to the well. These data were analyzed and shown as the area under the original curve (AUC).

### Western blot and immunohistochemistry assays

Western blot assays were performed as previously described.^56^ Cell or tissue lysate was separated by SDS-PAGE and transferred to nitrocellulose membrane (GE Healthcare, Madison, WI, USA). After incubation with the corresponding antibodies, membranes were visualized using the West-Dura detection system (Thermo Scientific, Waltham, MA, USA). The signal was collected by ImageQuant LAS 4000 mini (GE Health, USA). Immunohistochemistry assay of pancreas was performed as previously described.^10^

### Animal experiments

All animals received humane care and were raised at a relative humidity of 50% with a 12-h light–dark cycle at 20–25 °C and given *ad libitum* access to water and food. The animal-relevant protocols were approved by the Institutional Animal Care and Use Committees at Shanghai Institute of Materia Medica.

HFD/STZ-induced type 2 diabetic mice were constructed as described.^13, 57^ Briefly, 6-week-old C57/BL6 male mice were intraperitoneally injected with STZ (25 mg/kg/day) continuously for 5 days after feeding with HFD containing 58% fat for 4 weeks. To select diabetic mice, 6-h fasting plasma glucose was measured in the STZ-injected mice after 3 days. *db/db* male mice (BKS.Cg-*Dock7*^*m* +/+^ *Lepr*^*db*^/J) were from Jackson Laboratory (Sacramento, CA, USA). Both diabetic mice were assigned randomly to two groups by glucose level and body weight (*n*=8). Vehicle (2% DMSO and 8% Tween 80 dissolved in saline) or SP6616 (50 mg/kg/day) was administrated daily by intraperitoneal injection for 5 weeks. Fasting blood glucose level from 6-h fasted mice was measured weekly. OGTT (1.5 g/kg) was carried out on diabetic mice after fasted overnight at the fourth week. Glucose level was measured from tail blood at 0, 15, 30, 45, 60, 90 and 120 min. Meanwhile, the insulin release during OGTT was also detected. Blood sample was obtained from tail veins and serum insulin concentration was determined by AlphaLISA insulin kit (PerkinElmer). At the termination of the study, mice were killed and tissues were analyzed.

### Data analysis

Data were shown as means±S.E.M. Two-tailed unpaired *t*-test was performed for comparison of two groups and one-way ANOVA analysis for >2 groups by GraphPad Prism 5 software (GraphPad Software, La Jolla, CA, USA). Significant differences were shown as *P<0.05; **P<0.01; ***P<0.001.

## Abbreviations

T2DM: type 2 diabetes mellitus
GSIS: glucose-stimulated insulin secretion
MTT: 3-(4,5-dimethylthiazol-2-yl)-2,5-diphenyltetrazolium bromide
Kv: voltage-gated potassium
VDCCs: voltage-dependent Ca^2+^ channels
HFD: high-fat diet
STZ: streptozocin
ScTx-1: stromatoxin-1
Erk1/2: extracellular-regulated protein kinases 1/2
PKC: protein kinase C
PI3K: phosphatidylinositol 3-kinase
Akt: serine/threonine-specific protein kinase
XIAP: X-linked inhibitor of apoptosis protein
GAPDH: glyceraldehyde-3-phosphate dehydrogenase
OGTT: oral glucose tolerance test
HbA1c: glycated hemoglobin
KRB: Krebs–Ringer bicarbonate
FBS: fetal bovine serum
AUC: area under the curve
FoxO1: Forkhead box protein O1
