# Supplementary material for: SP6616 as a new Kv2.1 channel inhibitor efficiently promotes β-cell survival involving both PKC/Erk1/2 and CaM/PI3K/Akt signaling pathways
Source: Cell Death Dis. 2016 May 5;7(5):e2216–. doi: 10.1038/cddis.2016.119 (PMC4917657; doi:10.1038/cddis.2016.119)
Supplement: Supplementary Information [file cddis2016119x1.doc]

**SUPPLEMENTAL INFORMATION**

**SP6616 as a new Kv2.1 channel inhibitor efficiently promotes β-cell survival involving both PKC/Erk1/2 and CaM/PI3K/Akt signaling pathways**

TT Zhou, LL Quan, LP Chen, T Du, KX Sun, JC Zhang, L Yu, Y Li, P Wan, LL Chen, BH Jiang, LH Hu, J Chen, X Shen

This Supplemental Information includes:

Supplementary materials and methods

References

Supplementary Figure Legends

**Supplementary materials and methods**

**Materials**

Nifedipine were obtained from Sigma-Aldrich (USA). Antibodies against caspase 3, p-PKC, PKC, p-Erk1/2, Erk1/2 and Bcl2 were from Cell Signaling Technology (USA), and Glyceraldehyde-3-phosphate dehydrogenase (GAPDH) from Kangcheng Biotech (China).

**Plasmid**

The genes coding for Kv2.2 channel were synthesized by SangonBiotech (China) based on the sequence of GenBank (GeneID: NM_004770.2). The genes were inserted into the sites between EcoRI and XhoI in pcDNA3.1a plasmid.

**Electrophysiological recording assay**

The whole-cell patch clamp recordings were performed using cultured CHO-Kv2.1 cells at room temperature with Axopatch-200B amplifier (Molecular Devices) as described previously.^1^ 60~80% correction of series resistance and capacitance compensation were applied on Axon-200B amplifier as following:

1. Open the seal test measurement. After the pipette was dipped into bath solution, carefully adjust the fast and slow compensation controls to minimize the pipette capacitance transient.

2. After formed whole-cell recording mode, switch on the whole-cell parameter button, rotate the series resistance button to about 10 MΩ and whole-cell cap controls to about 10 pF ( β= 1 in whole-cell mode). Then rotate the prediction button to about 90%, set LAG time about 5 µs, and then very carefully increase the correction % to minimize slow capacitance transient. Usually when the correction reaches 60% percent, overshot current (saturation effect) is easily observed, then quickly adjust Fast pipette compensation to stabilize the seal test current and slightly adjust both series resistance and whole-cell cap buttons to minimize the slow transient current, at same time, slightly adjust the fast pipette capacitance to avoid over-shot. Usually 70% correction is OK for further recordings, sometime corrections could reach 80% or even high (according to the manual of AXOPATCH 200B). Once there are over-shots during the current recording, go back to check seal test measurement, to check if there are re-seals of whole-cell patching, and adjust the above three buttons to minimize fast and slow transient current.

3. After finish the recording of Kv currents, using high concentration inhibitors to completely inhibit the currents, and then record several trials with the same voltage protocol. Also record 3-5 trials from the empty cells with similar cell body size, under the same voltage protocol. All these trials could be useful for offline leak subtraction by using the trial subtraction in clamp-fit software.

**Western blot assay**

INS-832/13 cells were cultured in 12-well plates and treated with different concentrations of SP6616 and STZ (0.4 mM). Cell lysate was separated by SDS-PAGE and transferred to nitrocellulose membrane (GE Health, USA). After incubation with the corresponding antibodies, membranes were visualized using the West-Dura detection system (Thermo Scientific, USA). The signal was collected by ImageQuant LAS 4000 mini (GE Health, USA).

**Electrocardiography (ECG) assay**

All animals received humane care and were raised at a relative humidity of 50% with a 12 h light-dark cycle at 20-25°C and given ad libitum access to water and food. The animal-relevant protocols were approved by the Institutional Animal Care and Use Committees at Shanghai Institute of Materia Medica.

ECG assay was performed according to the published approach.[^2^](#_ENREF_1) Nine-week-old C57/BL6 male mice was distributed into 3 groups (n = 10-14 per group) to evaluate the cardiotoxicity of SP6616. Amiodarone hydrochloride (200 mg/kg) ^3^ group was used as positive control to estimate feasibility of the animal experiment. Briefly, mice were anesthetized and intraperitoneal injected vehicle (Vehicle group), 200 mg/kg amiodarone hydrochloride (AMD group) or 50 mg/kg SP6616 (SP6616 group) followed by insertion of electrodes in the right hind limb, right front limb, left hind limb. Three-minute electrocardiographic recordings were obtained before injection and after administration for 1h and 3h. The data was collected by Animal Bio Amp and PowerLab 8/30 instruments (ADInstruments, Bella Vista, Australia). The QT-interval was defined as the period from the Q-peak to the end of the T-peak in seconds and was analyzed using Chart 5 Pro software (ADInstruments, Bella Vista, Australia).

**References**

1. Li Y, Gamper N, Hilgemann DW, Shapiro MS. Regulation of Kv7 (KCNQ) K+ channel open probability by phosphatidylinositol 4,5-bisphosphate. *J Neurosci* 2005; **25:** 9825-9835.

2. Jiang B, Zhang L, Li M, Wu W, Yang M, Wang J *et al*. Salvianolic acids prevent acute doxorubicin cardiotoxicity in mice through suppression of oxidative stress. *Food Chem Toxicol* 2008; **46**: 1510-1515.

3. Lu WJ, Zhou J, Ma HY, Lu GH, You FQ, Ding AW *et al*. Effect of anti-arrhythmia drugs on mouse arrhythmia induced by Bufonis Venenum. *Yao xue xue bao* 2011; **46**: 1187-1192.

**Supplementary Figure legends**

**Supplementary** **Figure 1. SP6616 shows weak inhibition against Kv2.2 channel**

(a) pCDNA3.1a-Kv2.2-transfected CHO cells were incubated with 20 μM SP6616 or 100 nM stromatoxin-1 (ScTx-1) and membrane potential dye for 30min, and the signal was then collected. The data was analyzed and shown as area under the curve (AUC). (b) Membrane potential assay was conducted as (a) in CHO cells transfected with pCDNA3.1a. (c) IC_50_ of SP6616 in membrane potential assay was 13.48 μM. All data was obtained from three independent experiments and shown as means ± sem (**p<0.01, ***p<0.001).

**Supplementary** **Figure 2. SP6616 has no effects on p38, JNK or NFκB phosphorylation**

(a) INS-832/13 cells were incubated with SP6616 (10 μM) in the presence or absence of STZ (0.4 mM) for 24 h, and the cell lysate was analyzed by western blot assay using the corresponding antibodies. (b) Relative protein levels of p-p38/p38 in (a). (c) Relative protein levels of p-JNK/JNK in (a). (d) Relative protein levels of p-NFκB/NFκB in (a). All data was obtained from three independent experiments and presented as means ± sem (*p<0.05, ***p<0.001).

**Supplementary** **Figure 3. GFX increases phosphorylated Erk1/2 in INS-832/13 cells**

(a) INS-832/13 cells were separately incubated with GFX (1, 10, 20 μM) for 15 min, 1, 2, 16 or 24 h, and the cell lysate was analyzed by western blot assay using p-PKC, PKC, p-Erk1/2 and Erk1/2 antibodies. (b) Relative protein levels of p-PKC/PKC in (a). (c) Relative protein levels of p-Erk1/2/Erk1/2 in (a). All data was obtained from three independent experiments and presented as means ± sem (*p<0.05, **p<0.01, ***p<0.001).

**Supplementary Figure 4. Bcl-2 is not involved in the SP6616-mediated β-cell protection**

(a) INS-832/13 cells were incubated with various concentrations of SP6616 (1, 5, 10 μM) in the presence or absence of STZ (0.4 mM) for 24 h, and the cell lysate was analyzed by western blot using corresponding antibodies. (b) Relative protein levels of Bcl-2/GAPDH in (a). (c) INS-832/13 cells were incubated with various concentrations of Bcl-2 inhibitor ABT-263 (0.1, 0.5, 1, 10 μM) for 24 h and then MTT assay was conducted. ABT-263 did not affect the cell viability of INS-832/13 cells at the concentration of 0.1 or 0.5 μM. (d) INS-832/13 cells were incubated with SP6616 (10 μM) and STZ (0.4 mM) in the presence or absence of ABT-263 (0.1 μM) for 24 h, then MTT assay was performed. (e) INS-832/13 cells were incubated with SP6616 (10 μM) and STZ (0.4 mM) in the presence or absence of ABT-263 (0.5 μM) for 24 h, then MTT assay was carried out. All data was obtained from three independent experiments and presented as means ± sem (*p<0.05, **p<0.01, ***p<0.001).

**Supplementary Figure 5. Acute administration of SP6616 slightly prolongs the QT intervals without affecting heart rates in mice**

(a) The effects of AMD or SP6616 treatment on mouse heart rates in ECG assay. (b) The quantitative QT-interval duration of AMD and SP6616 treatment in mice. QT-interval was defined as the period from the Q-peak to the end of the T-peak in seconds. Values (n = 10-14 per group) were expressed as means ± sem (*p<0.05, **p<0.01, ***p<0.001).
