# Supplementary figures and images for: SP6616 as a new Kv2.1 channel inhibitor efficiently promotes β-cell survival involving both PKC/Erk1/2 and CaM/PI3K/Akt signaling pathways
Source: Cell Death Dis. 2016 May 5;7(5):e2216–. doi: 10.1038/cddis.2016.119 (PMC4917657; doi:10.1038/cddis.2016.119)

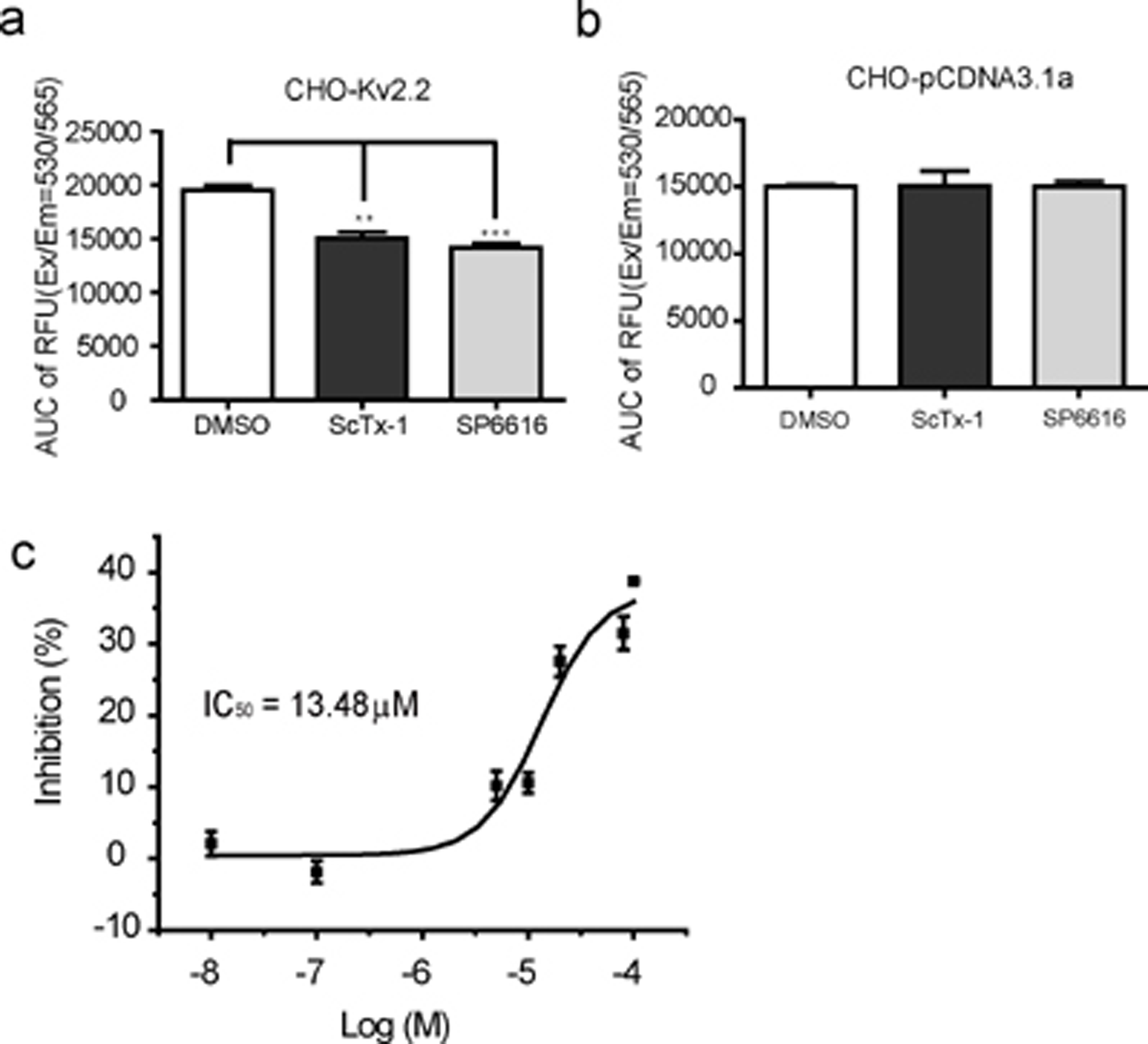

Supplement: Supplementary Figure 1 [file cddis2016119x2.tif]

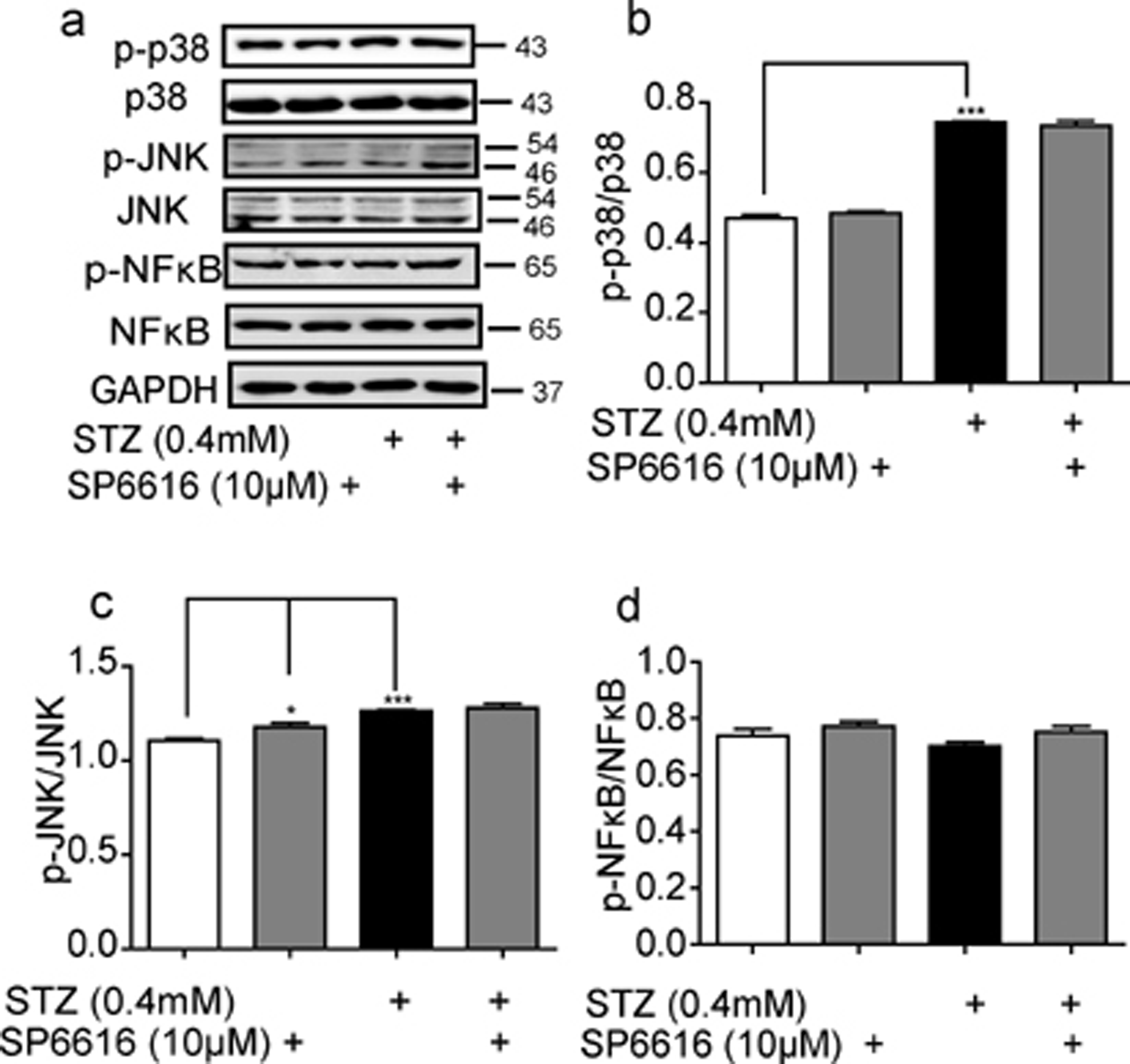

Supplement: Supplementary Figure 2 [file cddis2016119x3.tif]

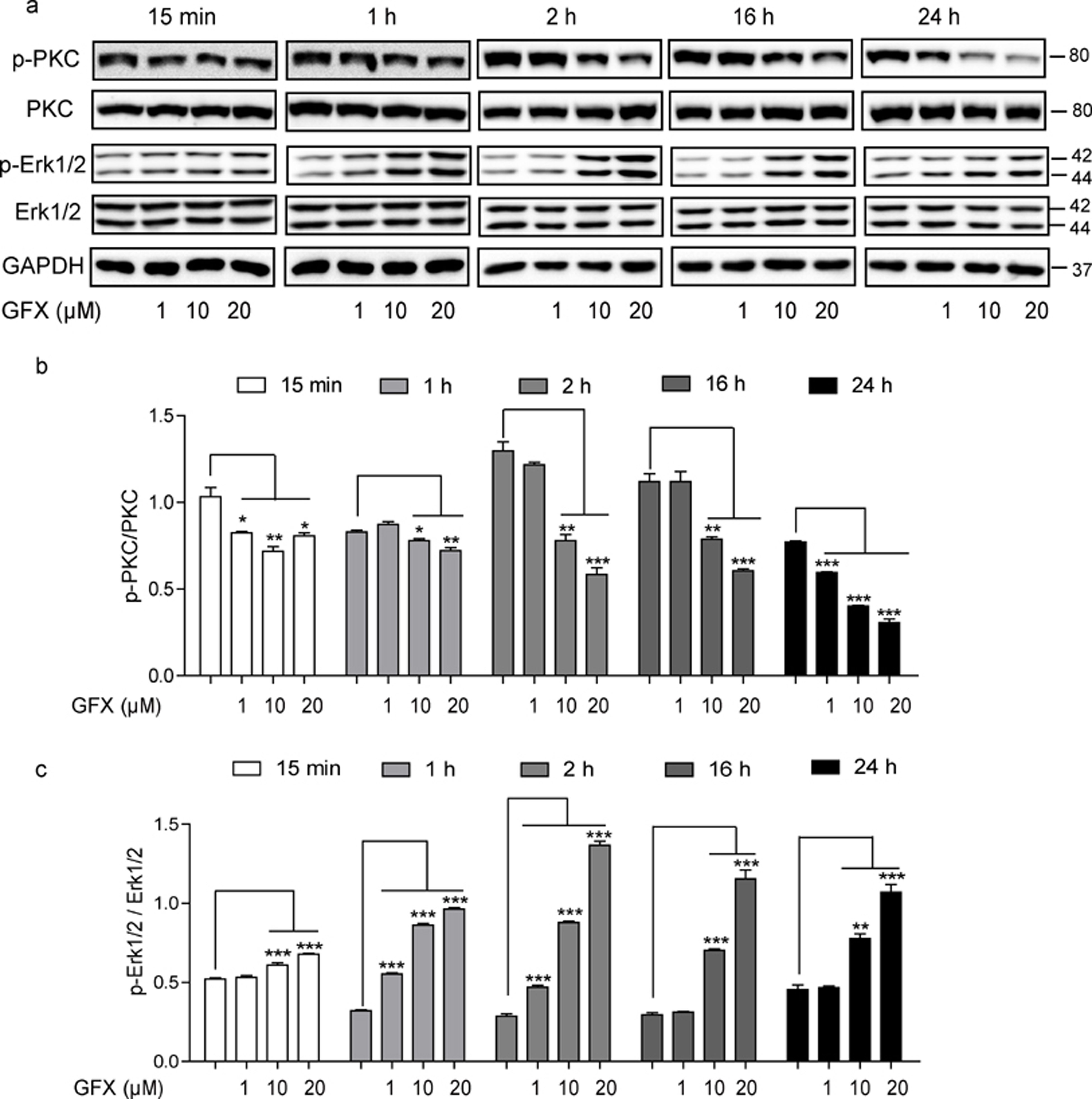

Supplement: Supplementary Figure 3 [file cddis2016119x4.tif]

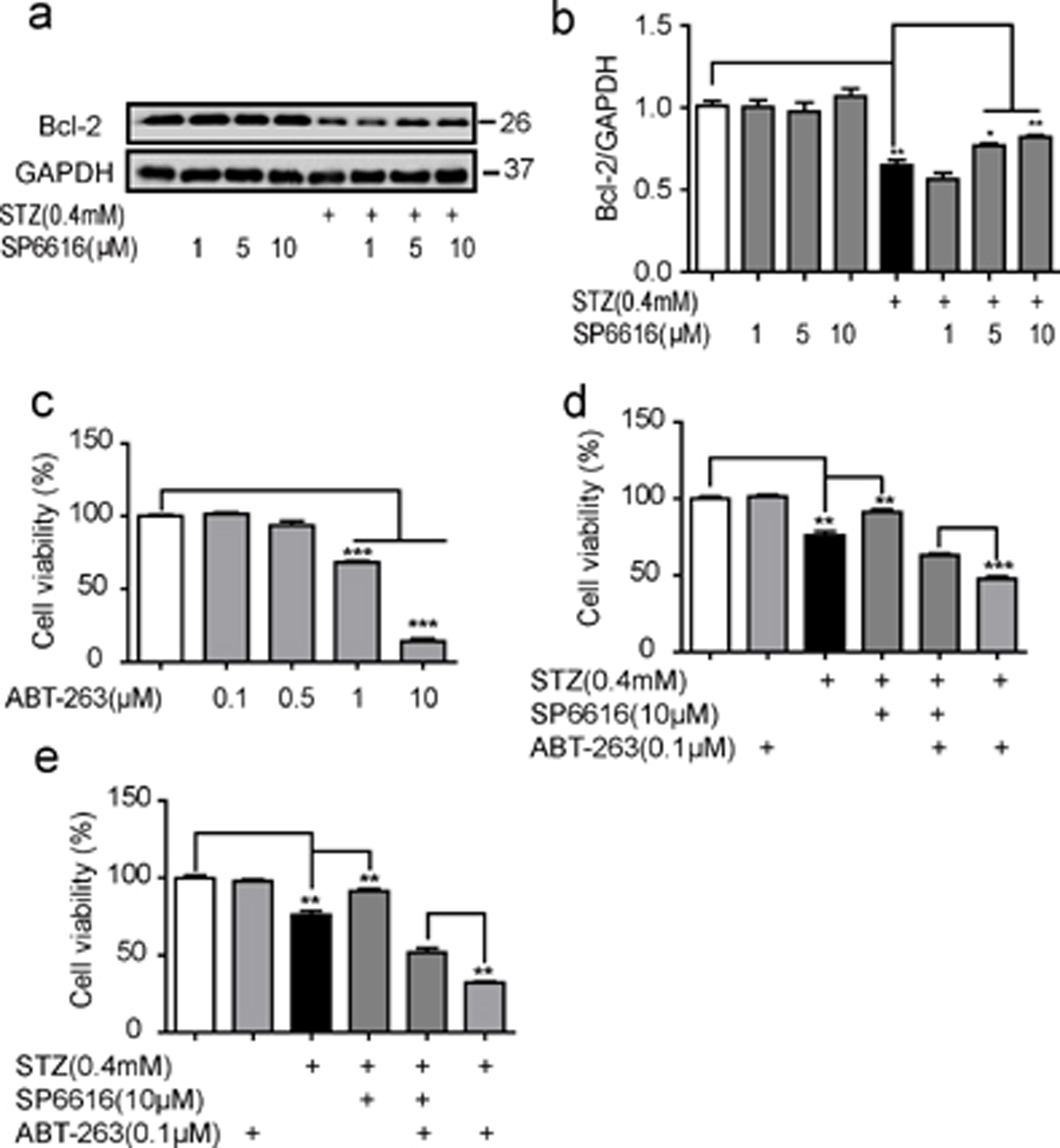

Supplement: Supplementary Figure 4 [file cddis2016119x5.tif]

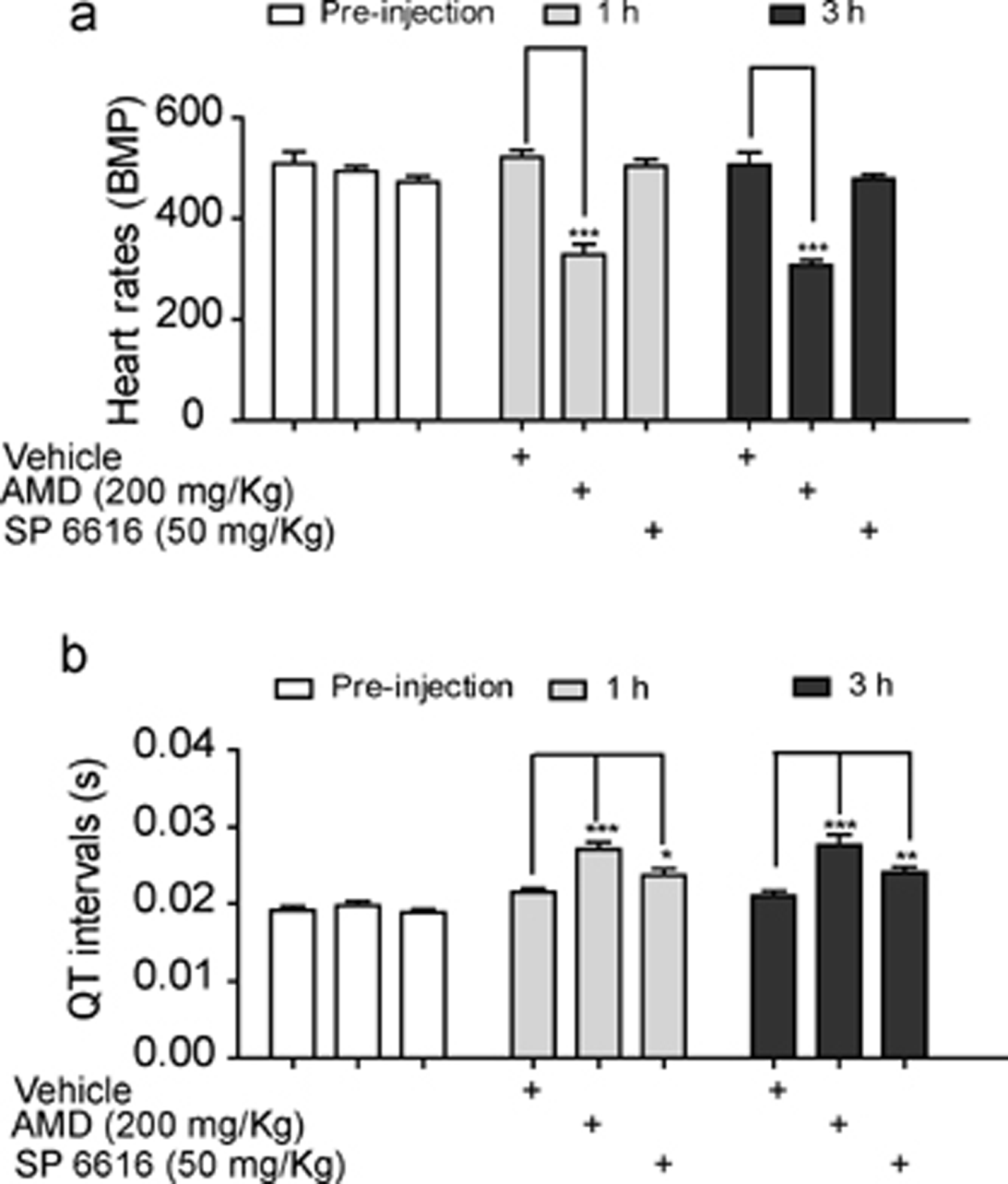

Supplement: Supplementary Figure 5 [file cddis2016119x6.tif]
